# Contrast in beliefs and knowledge about dementia risk reduction in Southern Latin American Indigenous and non‐Indigenous older adults: A qualitative analysis

**DOI:** 10.1002/alz.092368

**Published:** 2025-01-09

**Authors:** Jose M Aravena, Habana Muñoz, Daniela Vargas, Cecilia Albala, Becca R Levy

**Affiliations:** ^1^ Department of Social & Behavioral Sciences, School of Public Health, Yale University, New Haven, CT USA; ^2^ Yale University, New Haven, CT USA; ^3^ Villarrica Municipality, Villarrica Chile; ^4^ Universidad Católica del Maule, Curico Chile; ^5^ University of Chile, Santiago Chile

## Abstract

**Background:**

Although ethnic and cultural aspects can influence health behaviors, no studies have compared views about dementia and brain health between Indigenous and non‐Indigenous people living in the same territory. Therefore, we contrasted beliefs and knowledge about dementia risk reduction between Indigenous (Mapuche) and non‐Indigenous older adults in Chile.

**Method:**

An exploratory qualitative study was conducted with people 60 years and older, self‐identified as Mapuche (*‘people of the land’*) or non‐Mapuche, with no dementia. We recruited people living in urban and rural settings of Santiago and the Araucania region of Chile using a convenience and snowball strategy. Interviews covered topics such as cognitive changes of aging and dementia, dementia risk factors, and practices for brain health. Thematic analysis following grounded theory and inductive analysis were used to generate main topics.

**Result:**

A total of 24 in‐depth interviews (Mapuche: 14, non‐Mapuche: 10) were analyzed. For Mapuche people engaged in cultural religious practices, dementia was described as a *‘transition to their ancestors’ spiritual plane’*. Regarding potential risk factors of dementia, Mapuche people were more prone to refer to trauma and grief, low‐quality food, social problems, and alcoholism as major risk factors. Non‐Mapuche people were more likely to mention loneliness and depression, as major risk factors. Regarding positive elements for brain health, non‐Mapuche people highlighted participating in more activities, an *‘active attitude’*, social activities engagement, and cognitive activities. In contrast, Mapuche people prioritized *‘being in balance’* (referred to as avoiding any kind of *‘excess’*, or mind‐body and person‐environment connections), natural nutrition, good sleep, pride in being Mapuche, and engaging in traditional Mapuche activities like agriculture and handcrafts as ways to promote brain health.

**Conclusion:**

Even though groups share some common views about memory problems, there are differences in conceptions about the causes and prevention of dementia between Mapuche and non‐Mapuche older adults. Cultural and ethnic determinants may influence views and practices toward brain health and should be considered for designing dementia risk reduction interventions.

